# On eight species of jumping spiders from Xishuangbanna, Yunnan, China (Araneae, Salticidae)

**DOI:** 10.3897/zookeys.909.47137

**Published:** 2020-02-05

**Authors:** Cheng Wang, Shuqiang Li

**Affiliations:** 1 College of Agriculture and Forestry Engineering and Planning, Tongren University, Tongren 554300, Guizhou, China Tongren University Tongren China; 2 Institute of Zoology, Chinese Academy of Sciences, Beijing 100101, China Institute of Zoology, Chinese Academy of Sciences Beijing China

**Keywords:** Morphology, new species, salticid, South China, taxonomy

## Abstract

Seven new species of jumping spiders collected from Xishuangbanna Tropical Botanical Garden, China are diagnosed and described: *Cytaea tongi***sp. nov.** (♂♀), *Dexippus pengi***sp. nov.** (♂♀), *Euophrys subwanyan***sp. nov.** (♂♀), *Gelotia liuae***sp. nov.** (♂♀), *Irura lvshilinensis***sp. nov.** (♂♀), *Rhene menglunensis***sp. nov.** (♂♀), and *Siler zhangae***sp. nov.** (♂). The female of *Gelotia zhengi* Cao & Li, 2016 is described for the first time.

## Introduction

Of the 6173 jumping spider species known worldwide (WSC 2020), more than 500 species and nearly 125 genera have been recorded from China (Metzner 2020). Of these, at least 30 species in 26 genera have been described as new from Xishuangbanna in Yunnan, South China (Peng and Yin 1991; Song 1991; Peng 1995; Xie and Peng 1995; Peng and Kim 1997; Song and Zhu 1998; Xiao 2002; Xiao and Wang 2004; Cao and Li 2016). Despite the collecting conducted in the region, new species, typically known by only a single sex, are frequently discovered, which indicates that jumping spider fauna in Xishuangbanna is understudied, with the true diversity remaining elusive.

Recently, several expeditions to Xishuangbanna Tropical Botanical Garden (XTBG) were carried out by colleagues from the Chinese Academy of Sciences, and more jumping spiders were collected. In this paper, seven new species are described in addition to the female of *Gelotia zhengi* Cao & Li, 2016 for the first time.

## Material and methods

Specimens were mainly collected by fogging, beating shrubs, and hand collecting from the tree canopy, tree trunks, and leaf litter in the tropical rainforest of Xishuangbanna, Yunnan, China. All specimens were preserved in 75% ethanol. All specimens are deposited in the Institute of Zoology, Chinese Academy of Sciences (IZCAS) in Beijing, China.

The specimens were examined with an Olympus SZX16 stereomicroscope. After dissection, the epigyne was cleared in trypsin enzyme solution before examination and imaging. Left male palps were used for the description and illustration. Photos of the copulatory organs and habitus were taken with a Kuy Nice CCD mounted on an Olympus BX53 compound microscope. Compound focus images were generated using Helicon Focus v. 6.7.1.

All measurements are given in millimeters. Leg measurements are given as: total length (femur, patella + tibia, metatarsus, tarsus). References to figures in the cited papers are listed in lowercase type (fig. or figs); figures in this paper are noted with an initial capital (Fig. or Figs). Abbreviations used in the text and figures are as follows:

**AERW** anterior eye row width;

**ALE** anterior lateral eye;

**AME** anterior median eye;

**AR** anterior chamber of receptacle;

**BR** body of receptacle;

**C** conductor;

**CD** copulatory duct;

**CO** copulatory opening;

**CP** cymbial process;

**E** embolus;

**EFL** eye field length;

**FD** fertilization duct;

**F** fold;

**ICR** intermediate canal of receptacle;

**H** hood;

**HR** head of receptacle;

**PERW** posterior eye row width;

**PLE** posterior lateral eye;

**PME** posterior median eye;

**PR** posterior chamber of receptacle;

**PTA** prolateral tibial apophysis;

**RPA** retrolateral patella apophysis;

**RTA** retrolateral tibial apophysis;

**R** receptacle;

**S** septum;

**SD** sperm duct;

**VTA** ventral tibial apophysis;

**W** window.

## Taxonomy

### Family Salticidae Blackwall, 1841

#### Genus Cytaea

Taxon classificationAnimaliaAraneaeSalticidae

Keyserling, 1882

E09313A6-0EDA-590E-A326-38E509B2BC57

##### Type species.

*Cytaea alburna* Keyserling, 1882 from Australia.

##### Comments.

The genus *Cytaea* contains 41 nominal species and is currently known from the Asia and Oceania. It is rather poorly studied, as more than half (22) of its species are only known from a single sex and some species have no diagnostic illustrations and can not be confidently identified.

#### Cytaea tongi sp. nov.

Taxon classificationAnimaliaAraneaeSalticidae

0E9D96F4-1394-5745-BEF2-9D8D61BC61AB

http://zoobank.org/38F11BF9-1DFA-4059-AD56-76522CB34B79

**Figure 1.**
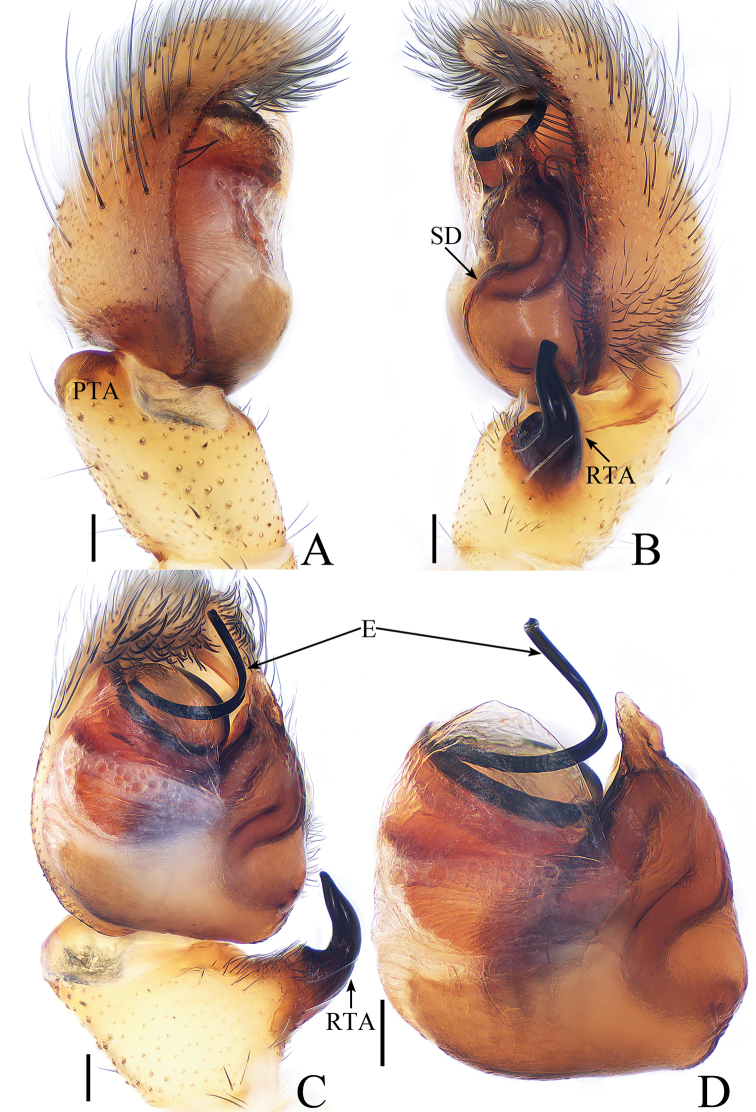
Male palp of *Cytaea tongi* sp. nov., **A**–**C** holotype; **D** paratype. **A** prolateral **B** retrolateral **C** ventral **D** bulb, ventral. Scale bars: 0.1.

**Figure 2.**
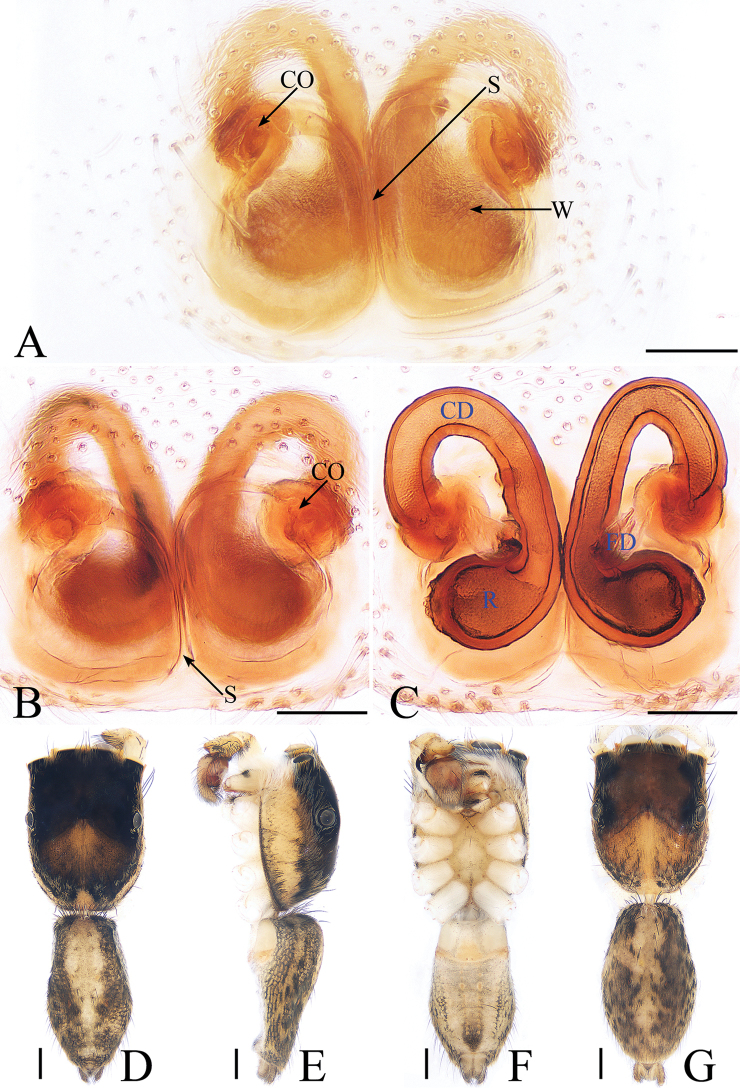
*Cytaea tongi* sp. nov., female paratype and male holotype. **A**, **B** epigyne, ventral **C** epigyne, dorsal **D** holotype habitus, dorsal **E** holotype habitus, lateral **F** holotype habitus, ventral **G** female paratype habitus, dorsal. Scale bars: 0.1 (**A**–**C**); 0.5 (**D**–**G**).

Figs 1 , 2 , 17A , 18A , 19A

##### Type material.

***Holotype*** ♂ (IZCAS Ar 39756) CHINA: Yunnan: Xishuangbanna, Mengla County, Menglun Town, Menglun Nature Reserve, Xishuangbanna Tropical Botanical Garden, tropical rainforest (21°55.20'N, 101°16.21'E, ca 550 m), 26.04.2019, Y.F. Tong et al. leg. ***Paratypes***: 2♂ 4♀ (IZCAS Ar 39757–39762), same data as holotype; 1♀ (IZCAS Ar 39763), garbage dump, secondary tropical rainforest (21°54.33'N, 101°16.79'E, ca 620 m), 7.05.2019, Y.F. Tong et al. leg; 1♂ (IZCAS Ar 39764), Leprosy Village (21°53.62'N, 101°18.25'E, ca 520 m), 29.04.2019, Y.F. Tong et al. leg; 1♀ (IZCAS Ar 39765), Leprosy Village (21°53.59'N, 101°17.30'E, ca 550 m), 4.05.2019, Y.F. Tong et al. leg.

##### Etymology.

The specific name is a patronym after Yanfeng Tong (Shenyang, China), one of the collectors of the new species.

##### Diagnosis.

*Cytaea tongi* sp. nov. resembles *C. oreophila* Simon, 1902 known from Indonesia and Singapore by the shape of the copulatory organs and habitus but differs in the following: 1) the RTA is curved towards the bulb medially in ventral view (Fig. 1C), whereas it is curved towards the bulb terminally in *C. oreophila* (Zhang and Maddison 2015, fig. 554); 2) the RTA is S-shaped and tapering in retrolateral view (Fig. 1B), whereas it is straight and broadening in *C. oreophila* (Zhang and Maddison 2015, fig. 555); 3) the epigynal window occupies about two-thirds of the epigynal plate (Fig. 2A, B), whereas it occupies more than nine-tenths of the plate in *C. oreophila* (Zhang and Maddison 2015, fig. 557). The male of *C. tongi* sp. nov. also resembles those of *C. carolinensis* Berry, Beatty & Prószyński, 1998, known from Caroline Island, by the shape of male palp but differs by the following: 1) the RTA is strongly curved medially in ventral view (Fig. 1C), whereas it is straight in *C. carolinensis* (Berry et al. 1998, fig. 8); 2) the tip of the RTA is blunt in retrolateral view (Fig. 1B), whereas it is pointed in *C. carolinensis* (Berry et al. 1998, fig. 9).

##### Description.

***Male*.** Total length 5.09. Carapace 2.46 long, 1.87 wide. Abdomen 2.52 long, 1.37 wide. Clypeus 0.14 high. Eye sizes and inter-distances: AME 0.46, ALE 0.32, PLE 0.28, AERW 1.83, PERW 1.87, EFL 1.15. Legs: I 6.11 (1.63, 2.66, 1.22, 0.60), II 5.55 (1.63, 2.15, 1.22, 0.55), III 5.81 (1.80, 1.95, 1.46, 0.60), IV 6.04 (1.90, 2.05, 1.49, 0.60). Carapace (Figs 2D–F, 17A) yellow-brown to dark-brown, with dense white and yellow scale-like setae around eyes, stripes of yellow setae posteriorly. Fovea longitudinal, situated between PLEs. Clypeus yellow, covered with dense white setae. Chelicerae (Fig. 18A) pale yellow with 5 promarginal teeth and 1 retromarginal fissident with 2 cusps. Endites, labium, and sternum colored as chelicerae. Legs pale yellow except dorsum of femora green. Spination of leg I: femur d1-1-5; patella p0-1-1, r0-1-0; tibia d1-0-0, p1-2-0, r1-2-0, v2-2-2; metatarsus p2-0-2, v2-0-2. Abdomen (Fig. 2D–F) elongated oval, dorsum with 2 pairs of muscle depressions medially, irregular pale yellow stripe nearly extending across the entire surface and bifurcated posteriorly, covered with dense brown setae and sparse, long setae; venter pale brown, with 2 rows of spots medially and a large dark spot close to the spinnerets. Palp (Fig. 1A–D): femur yellow, about 2.5 times longer than wide, covered with setae; patella colored as femur, almost as long as wide, covered with white setae; tibia stocky, slightly wider than long, with lobe-shaped prolateral apophysis and sclerotized, hook-shaped RTA curved towards bulb medially; cymbium longer than wide, covered with dense setae; bulb approximately as long as wide, retrolatero-terminally with 2 round processes; embolus long, completing nearly full flattened coil at base, the base of the embolus mostly hidden by membranous structure on bulb, with blunt tip that reaches cymbial tip.

***Female*.** Total length 5.26. Carapace 2.41 long, 1.91 wide. Abdomen 2.63 long, 1.54 wide. Clypeus 0.15 high. Eye sizes and inter-distances: AME 0.46, ALE 0.32, PLE 0.27, AERW 1.83, PERW 1.87, EFL 1.15. Legs: I 5.31 (1.59, 2.12, 1.05, 0.55), II 4.75 (1.59, 1.66, 0.95, 0.55), III 5.33 (1.63, 1.88, 1.27, 0.55), IV 5.50 (1.71, 1.90, 1.34, 0.55). Habitus (Fig. 2G) similar to those of male except paler. Epigyne (Fig. 2A–C) almost as long as wide, windows large, separated by narrow septum; copulatory openings almost round, situated latero-medially; copulatory ducts extremely short, inverse U-shaped; receptacles oval, about 1.5 times the diameter of the copulatory ducts; fertilization ducts membranous, lamellar.

##### Distribution.

China (Yunnan).

##### Comments.

Although differing greatly from the type species of the genus, we place the new species in *Cytaea* because it is similar to *C. oreophila* and *C. carolinensis*, two species already placed in this genus.

#### Dexippus

Taxon classificationAnimaliaAraneaeSalticidae

Thorell, 1891

580225E2-73E4-5799-88C6-88BF85B190C1

##### Type species.

*Dexippus kleini* Thorell, 1891 from Indonesia.

##### Comments.

The poorly known genus *Dexippus* contains three species, one endemic each to Indonesia, India, and China. Two are known from males, and *D. topali*Prószyński 1992 is known from both sexes. There are three papers that provide diagnostic illustrations of the type species and descriptions of the two other species (Prószyński 1984, 1992; Peng and Li 2002).

#### Dexippus pengi sp. nov.

Taxon classificationAnimaliaAraneaeSalticidae

130DD18D-6124-540F-A533-CAF2852DF69D

http://zoobank.org/06321D4E-4D79-4385-AC9A-0AFFA338983F

**Figure 3.**
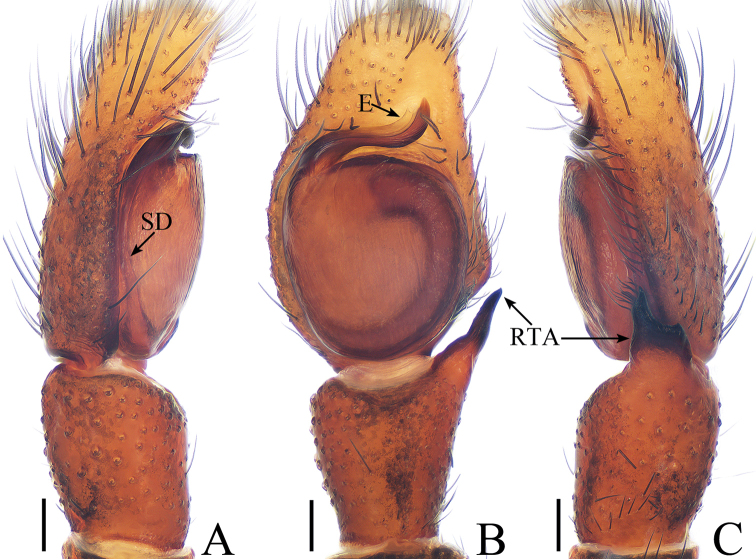
Male palp of *Dexippus pengi* sp. nov., holotype. **A** prolateral **B** ventral **C** retrolateral. Scale bars: 0.1.

**Figure 4.**
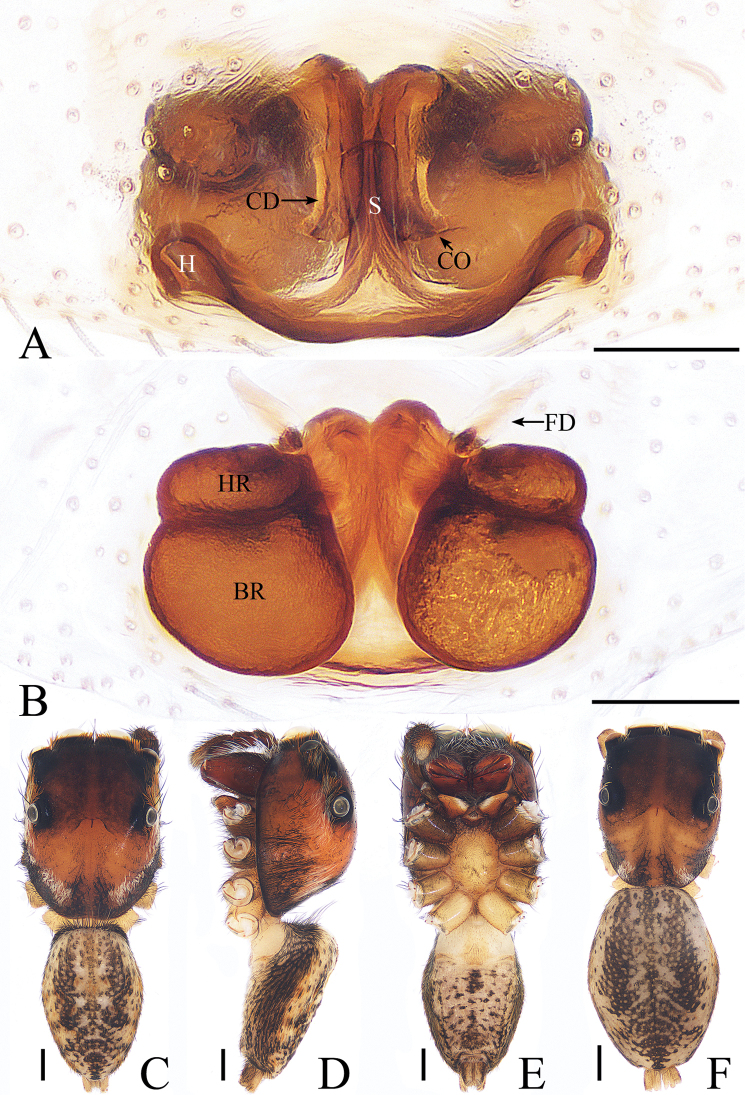
*Dexippus pengi* sp. nov., female paratype and male holotype. **A** epigyne, ventral **B** epigyne, dorsal **C** holotype habitus, dorsal **D** holotype habitus, lateral **E** holotype habitus, ventral **F** female paratype habitus, dorsal. Scale bars: 0.1 (**A**–**B**); 0.5 (**C**–**F**).

Figs 3 , 4 , 17C , 18C , 19C

##### Type material.

***Holotype*** ♂ (IZCAS Ar 39771) CHINA: Yunnan: Xishuangbanna, Mengla County, Menglun Town, Menglun Nature Reserve, Mannanxing Village (21°53.49'N, 101°17.12'E, ca 560 m), 9.08.2018, C. Wang et al. leg. ***Paratypes***: 2♂ 2♀ (IZCAS Ar 39772–39775), same data as holotype; 2♀ (IZCAS Ar 39776–39777), same locality, tropical rainforest (21°55.35'N, 101°16.36'E, ca 610 m), 7.08.2018, C. Wang et al. leg; 1♂ 1♀ (IZCAS Ar 39778–39779), same locality, tropical rainforest (21°55.05'N, 101°16.24'E, ca 570 m), 26.07.2018, X.Q. Mi et al. leg; 1♂ (IZCAS Ar 38780), Leprosy Village (21°53.59'N, 101°17.30'E, ca 550 m), 4.05.2019, Y.F. Tong et al. leg; 2♂ (IZCAS Ar 39781–39782), same locality, Vine Garden (21°55.80'N, 101°45.41'E, ca 550 m), 2.08.2018, C. Wang et al. leg; 1♂ 3♀ (IZCAS Ar 39783–39786), same locality, tropical rainforest (21°55.20'N, 101°16.21'E, ca 550 m), 30.04.2019, Y.F. Tong et al. leg.

##### Etymology.

The specific name is a patronym in honor of Dr Xianjin Peng (Changsha, China), who has produced many important works on the taxonomy of Chinese jumping spiders.

##### Diagnosis.

*Dexippus pengi* sp. nov. resembles *D. topali* Prószyński, 1992 from India by the shape of the copulatory organs and habitus but differs in the following: 1) palpal tibia is longer than wide (Fig. 3B–C), whereas it is wider than long in *D. topali* (Prószyński 1992, figs 12, 13); 2) the dorsal ramus of the RTA is thorn-like in retrolateral view (Fig. 3C), whereas it is not developed in *D. topali* (Prószyński 1992, fig. 13); 3) in the female, the copulatory openings are separated by a septum (Fig. 4A), whereas they are covered by a bell-shaped structure in *D. topali* (Prószyński 1992, fig. 14).

##### Description.

***Male*.** Total length 5.37. Carapace 2.76 long, 2.17 wide. Abdomen 2.41 long, 1.61 wide. Clypeus 0.09 high. Eye sizes and inter-distances: AME 0.57, ALE 0.37, PLE 0.35, AERW 2.07, PERW 2.07, EFL 1.30. Legs: I 5.10 (1.63, 2.05, 0.83, 0.59), II 4.62 (1.24, 2.01, 0.78, 0.59), III 5.25 (1.73, 1.88, 1.05, 0.59), IV 5.69 (1.90, 1.93, 1.27, 0.59). Carapace (Figs 4C–E, 17C) orange-brown, cephalic part darker, clothed with dense setae antero-bilaterally, thoracic part sloping abruptly, clothed with orange-brown and dark setae around eyes. Fovea longitudinal. Clypeus orange-brown to dark brown, covered with thin setae. Chelicerae (Fig. 18C) red-brown, with 1 retromarginal tooth and 2 promarginal teeth. Endites red-brown, inner tip pale. Labium dark brown, tip pale and covered with dark setae. Sternum yellow, covered with dark and grey-white setae. Legs red-brown, patella and tibia I with scopulae, legs III, IV paler. Spination of leg I: femur d0-1-5; patella p0-1-0; tibia d1-0-0, p1-1-0, r1-1-0, v2-2-2; metatarsus p0-0-1, v2-0-2. Abdomen (Fig. 4C–E) elongated oval, dorsum with 2 pairs of muscle depressions, irregular black-brown stripes, several chevrons postero-medially; venter pale brown, with dark spots. Palp (Fig. 3A–C): femur red-brown, about 3.3 times longer than wide, covered with dense setae; patella red-brown, slightly longer than wide; tibia distinctly longer than wide, RTA bifurcated with ventral ramus well-developed, tapering to a pointed tip, dorsal ramus thorn-like; cymbium flattened, covered with long setae; bulb almost round, with sperm duct extending along margin; embolus stout, originating near 10 o’clock position of bulb.

***Female*.** Total length 4.77. Carapace 2.21 long, 1.75 wide. Abdomen 2.60 long, 1.87 wide. Clypeus 0.09 high. Eye sizes and inter-distances: AME 0.55, ALE 0.31, PLE 0.27, AERW 1.69, PERW 1.69, EFL 1.06. Legs: I 3.78 (1.17, 1.56, 0.61, 0.44), II 3.76 (1.22, 1.49, 0.61, 0.44), III 4.60 (1.59, 1.54, 0.93, 0.54), IV 4.91 (1.59, 1.76, 1.02, 0.54). Habitus (Fig. 4F) similar to those of male except paler. Epigyne (Fig. 4A, B) wider than long, with pair of hoods near epigastral furrow; copulatory openings situated medially, separated by anchor-shaped septum; copulatory ducts relatively stout, ascending before extending almost transversely to connect with receptacles; receptacles divided into oval head and body.

##### Distribution.

China (Yunnan).

#### Euophrys

Taxon classificationAnimaliaAraneaeSalticidae

C.L. Koch, 1834

8BD2DCA1-8D93-50BD-83B3-DD97D5C6D2AE

##### Type species.

*Aranea frontalis* Walckenaer, 1802 from France.

##### Comments.

The genus *Euophrys* is one of the largest genera of the family Salticidae, currently containing 108 nominal species from the Holarctic, Afrotropical, and Neotropical realms (Prószyński et al. 2018; WSC 2020). The genus is rather poorly studied with 57 species only known from a single sex; some poorly known species have no diagnostic illustrations, and many species are pending re-classification (Prószyński et al. 2018; WSC 2020). To date, 34 species have been recorded from Asia. Of these, 19 species are known from only a single sex: eight males and 11 females. Seven species lack diagnostic illustrations, and one species is known from a description of a juvenile specimen. Presently, 12 species from China have diagnostic illustrations, including seven endemics. Five of these are known from only a single sex (WSC 2020).

#### Euophrys subwanyan sp. nov.

Taxon classificationAnimaliaAraneaeSalticidae

7443357A-1E9B-5527-BE20-1D8359218D97

http://zoobank.org/7F4D8CE5-556C-4781-B226-D6017DB7BF7B

**Figure 5.**
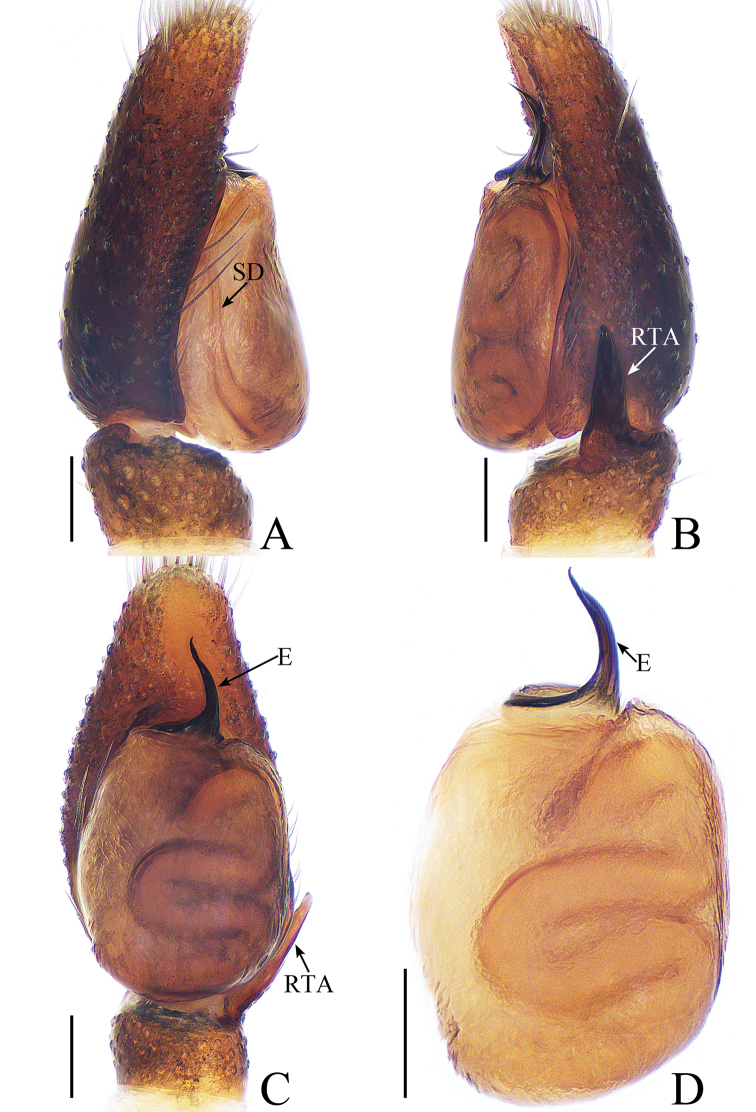
Male palp of *Euophrys subwanyan* sp. nov., **A**–**C** holotype **D** paratype **A** prolateral **B** retrolateral **C** ventral **D** bulb, ventral. Scale bars: 0.1.

**Figure 6.**
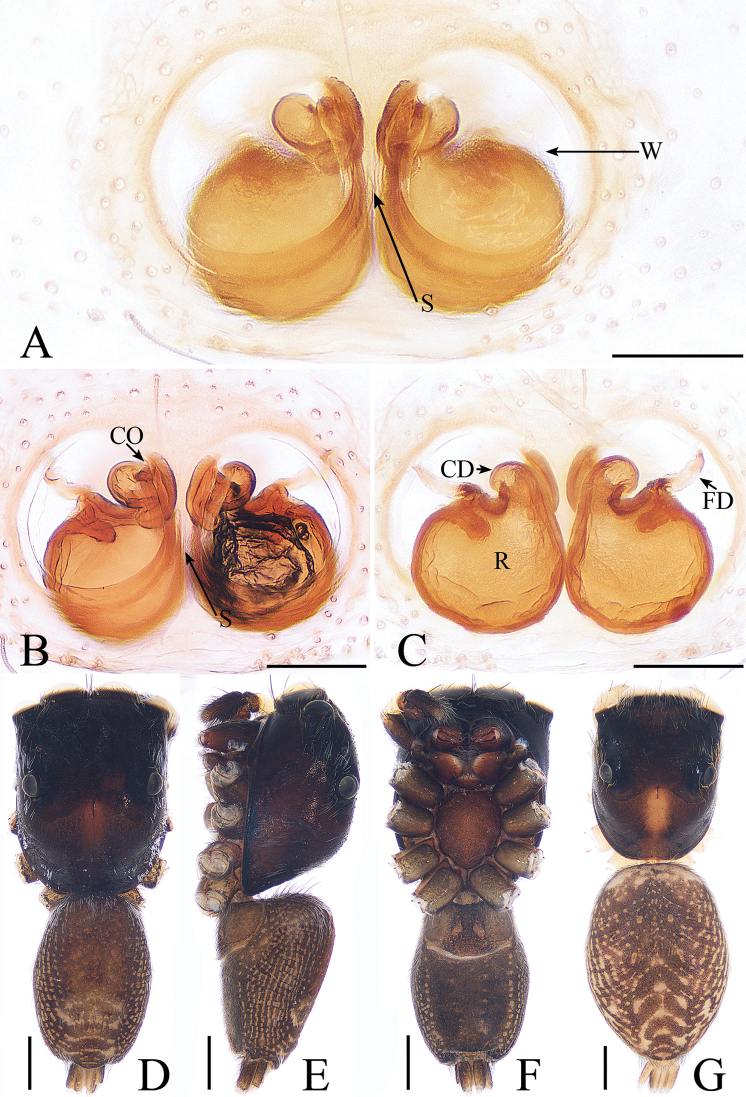
*Euophrys subwanyan* sp. nov., female paratype and male holotype. **A**, **B** epigyne, ventral **C** epigyne, dorsal **D** holotype habitus, dorsal **E** holotype habitus, lateral **F** holotype habitus, ventral **G** female paratype habitus, dorsal. Scale bars: 0.1 (**A**–**C**); 0.5 (**D**–**G**).

Figs 5 , 6 , 17B , 18B , 19B

##### Type material.

***Holotype*** ♂ (IZCAS Ar 39766) CHINA: Yunnan: Xishuangbanna, Mengla County, Menglun Town, Menglun Nature Reserve, Xishuangbanna Tropical Botanical Garden, Vine Garden (21°55.80'N, 101°45.41'E, ca 550 m), 2.08.2018, C. Wang et al. leg. ***Paratypes***: 1♂ 3♀ (IZCAS Ar 39767–39770), same data as holotype.

##### Etymology.

The specific epithet is referring to the similarity with *E. wanyan* Berry, Beatty & Prószyński, 1996, substantive.

##### Diagnosis.

*Euophrys subwanyan* sp. nov. resembles *E. wanyan* known from Caroline Island in the Eastern Pacific by the shape of the copulatory organs and habitus but differs by the following: 1) the embolus is directed anteriorly (Fig. 5C), whereas it is directed towards the cymbial prolateral margin in *E. wanyan* (Berry et al. 1996, fig. 55); 2) the tip of the RTA is directed anteriorly (Fig. 5C), whereas it is directed prolaterally in *E. wanyan* (Berry et al. 1996, fig. 55); 3) the copulatory ducts are coiled in a 360° spiral (Fig. 6C), whereas they are coiled in a 150° spiral in *E. wanyan* (Berry et al. 1996, fig. 58).

##### Description.

***Male*.** Total length 3.46. Carapace 1.86 long, 1.38 wide. Abdomen 1.67 long, 1.11 wide. Clypeus 0.07 high. Eye sizes and inter-distances: AME 0.43, ALE 0.29, PLE 0.25, AERW 1.48, PERW 1.33, EFL 0.92. Legs: I 3.61 (1.10, 1.37, 0.63, 0.51), II 3.05 (0.93, 1.12, 0.54, 0.46), III 3.49 (1.10, 1.15, 0.73, 0.51), IV 3.74 (1.20, 1.27, 0.76, 0.51). Carapace (Figs 6D–F, 17B) dark brown, cephalic part almost square, thoracic part sloping abruptly, bilaterally with scattered white setae. Fovea longitudinal, bar-shaped. Clypeus dark. Chelicerae (Fig. 18B) red-brown, with 2 promarginal teeth and 1 retromarginal tooth. Endites, labium and sternum colored as chelicerae. Sternum slightly longer than wide, covered with dark setae. Legs yellow to brown. Spination of leg I: femur d1-1-1; tibia v2-2-2; metatarsus p1-0-0, v2-0-2. Abdomen (Fig. 6D–F) elongated oval, speckled bilaterally, dorsum with a scutum covering anterior half, 2 pairs of muscle depressions located medially, and several chevrons posteriorly, covered with white setae, denser at anterior margin; venter dark brown, with 4 rows of spots. Palp (Fig. 5A–D): femur red-brown, about 3 times longer than wide; patella yellow, slightly longer than wide; tibia wider than long, with relatively long RTA slightly longer than tibia in retrolateral view, tapering to a slightly pointed tip; cymbium red-brown, longer than wide, widest medially; bulb longer than wide, with sperm duct relatively stout, meandering retrolaterally and tapering prolaterally; embolus with a coiled base that is perpendicular to the long axis of the palp, slightly curved medially, tip of embolus directed anteriorly.

***Female*.** Total length 4.18. Carapace 1.82 long, 1.43 wide. Abdomen 2.29 long, 1.63 wide. Clypeus 0.07 high. Eye sizes and inter-distances: AME 0.47, ALE 0.33, PLE 0.28, AERW 1.83, PERW 1.87, EFL 1.15. Legs: I 3.70 (1.10, 1.41, 0.63, 0.56), II 3.39 (1.12, 1.17, 0.59, 0.51), III 3.93 (1.24, 1.34, 0.83, 0.52), IV 4.34 (1.32, 1.46, 1.02, 0.54). Habitus (Fig. 6G) similar to that of male except paler. Epigyne (Fig. 6A–C) slightly wider than long, windows large, separated by narrow septum; copulatory openings on each side of septum located anteriorly; copulatory ducts curved anteriorly, then coiled 360° to connect with anterior edge of the receptacles; receptacles spherical, touching medially; fertilization ducts originating from the median anterior edge of receptacles, extending almost transversely.

##### Distribution.

China (Yunnan).

##### Comments.

The new species has been assigned to this genus due to similarity to *E. wanyan*. However, both species differ from the type species, *E. frontalis* (Walckenaer, 1802) (i.e. the face without coloured eyebrows, versus distinct eyebrows in *E. frontalis*; embolic base perpendicular to the long axis of the palp, versus parallel to the long axis of the palp in *E. frontalis*; RTA is not needle-shaped). Prószyński et al. (2018) doubted the placement *E. wanyan* in *Euophrys* and listed it as “*Euophrys*[?] *wanyan*”. Therefore, the generic placement of the new species is provisional.

#### Gelotia

Taxon classificationAnimaliaAraneaeSalticidae

Thorell, 1890

3C61B983-1B03-5B29-AFE7-C7AC8DE76605

##### Type species.

*Gelotia frenata* Thorell, 1890 from Indonesia.

##### Comments.

The genus *Gelotia* contains nine nominal species currently known from East and South Asia, peninsular Malaysia through the Indonesian archipelago to New Guinea (Wijesinghe 1991; WSC 2020). All species are endemic and each is known from a single country, except for *G. syringopalpis* Wanless, 1984, which is distributed in China, Malaysia, and Borneo. Although the genus was revised by Wanless (1984) and all species have diagnostic illustrations, six species, including the generotype, are known from only a single sex, indicating that *Gelotia* remains inadequately studied. To date, six species are recorded from Southeast Asia and only two, *G. syringopalpis* and *G. zhengi* Cao & Li, 2016, from China (WSC 2020).

#### Gelotia liuae sp. nov.

Taxon classificationAnimaliaAraneaeSalticidae

78F11DDD-2FFA-5B77-A5AF-301C25105D45

http://zoobank.org/DFE83826-81FE-49AF-8AA1-1C07AE506308

**Figure 7.**
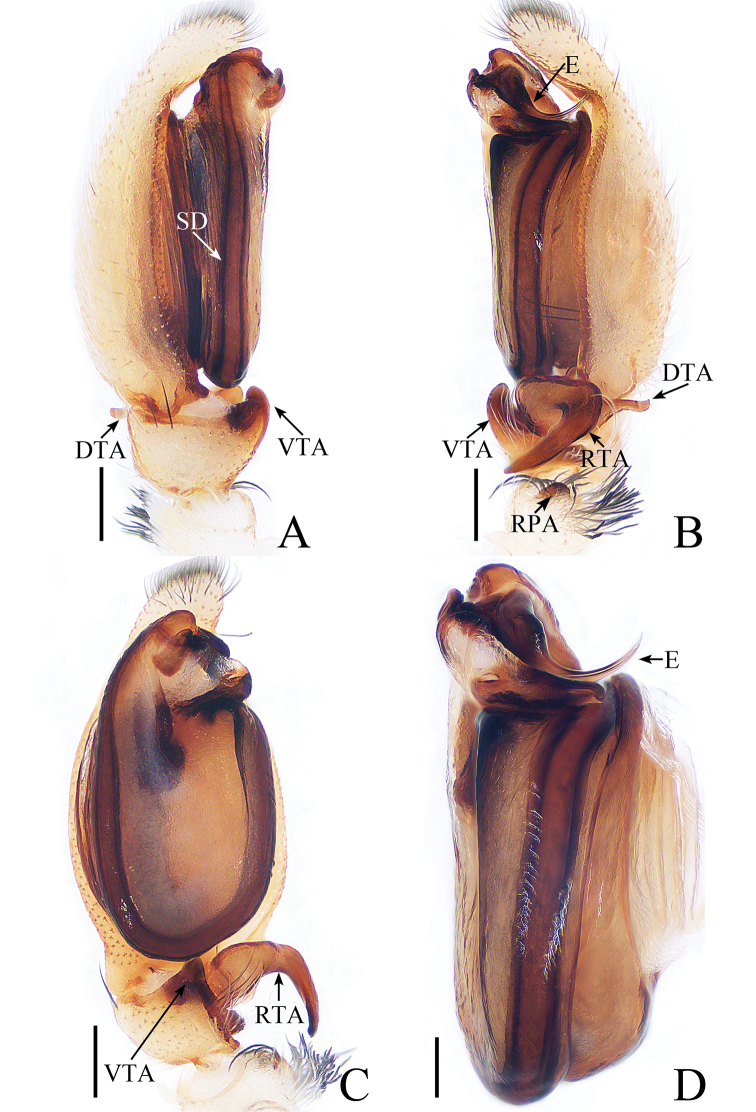
Male palp of *Gelotia liuae* sp. nov., **A**–**C** male holotype; **D** male paratype. **A** prolateral **B** retrolateral **C** ventral **D** bulb, retrolateral. Scale bars: 0.2 (**A**–**C**) ; 0.1 (**D**).

**Figure 8.**
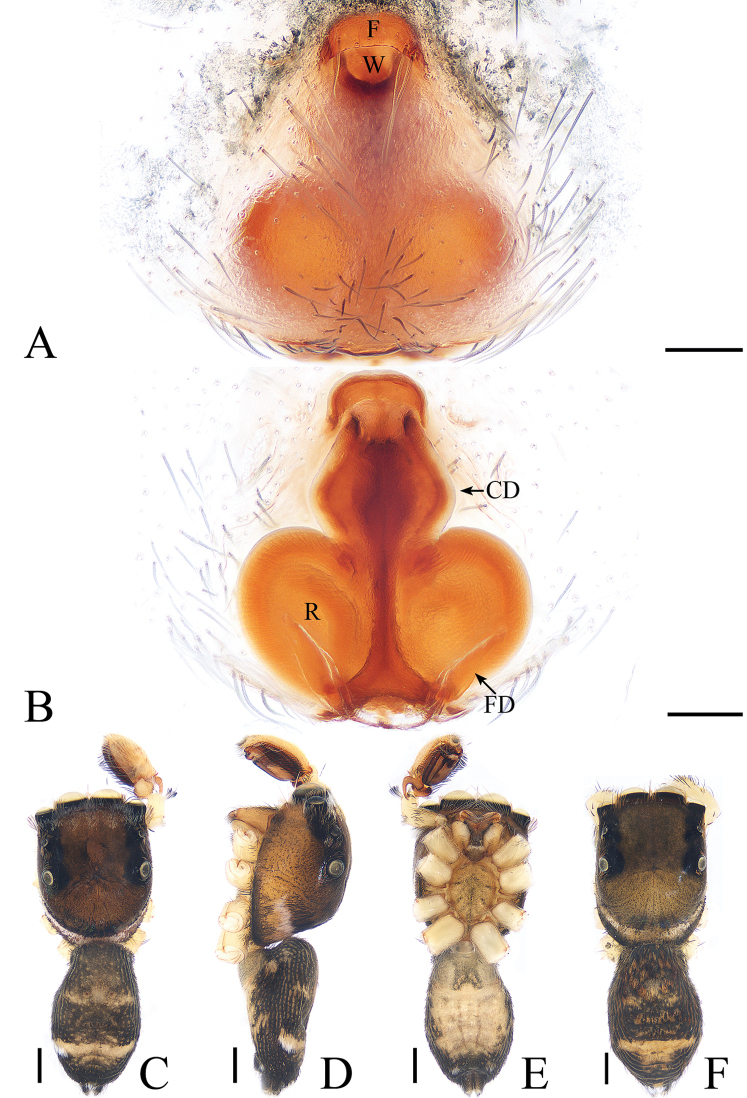
*Gelotia liuae* sp. nov., female paratype and male holotype. **A** epigyne, ventral **B** epigyne, dorsal **C** holotype habitus, dorsal **D** holotype habitus, lateral **E** holotype habitus, ventral **F** female paratype habitus, dorsal. Scale bars: 0.1 (**A**, **B**); 0.5 (**C**–**F**).

Figs 7 , 8 , 17D , 18D , 19D

Gelotia sp.: Maddison et al. 2014: 68, fig. 7 (♂).

##### Type material.

***Holotype*** ♂ (IZCAS Ar 39787) CHINA: Yunnan: Xishuangbanna, Mengla County, Menglun Town, Menglun Nature Reserve, Xishuangbanna Tropical Botanical Garden, tropical rainforest (21°55.20'N, 101°16.21'E, ca 550 m), 30.04.2019, Y.F. Tong et al. leg. ***Paratypes***: 3♂ 2♀ (IZCAS Ar 39788–39792), same data as holotype; 1♀ (IZCAS Ar 39793), tropical rainforest (21°55.20'N, 101°16.21'E, ca 550 m), 5.08.2018, C. Wang et al. leg; 1♀ (IZCAS Ar 39794), tropical rainforest (21°55.05'N, 101°16.24'E, ca 570 m), 26.07.2018, X.Q. Mi et al. leg.

##### Etymology.

The specific name is a patronym after Shijia Liu (Shenyang, China), one of the collectors of the new species.

##### Diagnosis.

The male of *G. liuae* sp. nov. resembles *G. syringopalpis*, known from Southeast Asia, in having 3 palpal tibial apophyses, a slender embolus, and a flattened tegulum but differs in the following: 1) the RTA is directed towards 7:30 o’clock in retrolateral view (Fig. 7B), whereas it is directed towards about 6 o’clock in *G. syringopalpis* (Wanless 1984, fig. 21D); 2) the dorsal tibial apophysis is obscured in ventral view and directed towards 3 o’clock in retrolateral view (Fig. 7B, C), whereas it is conspicuous and directed towards 1 o’clock in *G. syringopalpis* (Wanless 1984, fig. 21D, I). The female of the new species resembles *G. frenata* from Indonesia by the epigyne having a similar anterior sclerotized fold but differs in the following: 1) the receptacle is distant from the epigastral fold, the distance between them about half the length of the receptacle in dorsal view (Fig. 8B), whereas they are near the epigastral fold in *G. frenata*, with the distance between them less than one-tenth the length of the receptacle (Prószyński 1969, fig. 7); 2) the copulatory ducts width are about one-third the width of the receptacle (Fig. 8B), whereas the ducts are less than one-eighth the width of the receptacle in *G. frenata* (Prószyński 1969, fig. 7).

##### Description.

***Male*.** Total length 4.04. Carapace 2.08 long, 1.67 wide. Abdomen 2.12 long, 1.27 wide. Clypeus 0.10 high. Eye sizes and inter-distances: AME 0.49, ALE 0.29, PLE 0.25, AERW 1.62, PERW 1.59, EFL 1.08. Legs: I 5.13 (1.49, 1.88, 1.15, 0.61), II 4.78 (1.41, 1.68, 1.10, 0.59), III 4.59 (1.34, 1.56, 1.10, 0.59), IV 6.33 (1.80, 2.12, 1.80, 0.61). Carapace (Figs 8C–E, 17D) brown, darker in eye field, cephalic area almost square, covered with setae around eyes, thoracic part sloping acutely, with posterior marginal cambered stripes of white and black setae. Fovea longitudinal. Clypeus yellow, the anterior margin with long setae. Chelicerae (Fig. 18D) yellow, with 3 promarginal and 5 retromarginal teeth. Endites yellow. Labium brown. Sternum colored as endites, covered with sparse setae. Legs yellow to brown. Spination of leg I: femur d1-1-4; patella p0-1-0, r0-1-0; tibia d1-0-0, p0-1-1, r0-1-1, v2-2-2; metatarsus p1-1-0, r1-1-0, v2-0-2. Abdomen (Fig. 8C–E) elongated oval, dorsum speckled, with 2 pairs of muscle depressions medially, 2 transverse yellow stripes postero-medially; venter pale yellow, with 2 white dots close to the spinnerets. Palp (Fig. 7A–D): femur yellow, about 3 times longer than wide, covered with dense setae; patella pale yellow, with dense setae dorsally, tubelike retrolateral apophysis bearing long curved setae; tibia almost as long as wide, with ventral apophysis subtriangular in ventral view, RTA curved medially, directed towards 7 o’clock apically in retrolateral view, dorsal apophysis widest at base, extending transversely, blunt distally; cymbium flattened, narrowed posteriorly; bulb flattened, distally with well-developed lobe, sperm duct extending along margin, almost U-shaped; embolus originating from anterior edge of bulb, broadening at base, curved towards alveolus and pointed apically.

***Female*.** Total length 4.61. Carapace 2.39 long, 1.78 wide. Abdomen 2.33 long, 1.52 wide. Clypeus 0.11 high. Eye sizes and inter-distances: AME 0.50, ALE 0.30, PLE 0.25, AERW 1.74, PERW 1.75, EFL 1.15. Legs: I 5.35 (1.63, 2.01, 1.10, 0.61), II 5.02 (1.63, 1.76, 1.02, 0.61), III 4.82 (1.63, 1.56, 1.02, 0.61), IV 6.32 (1.83, 2.17, 1.71, 0.61). Habitus (Fig. 8F) similar to those of male except paler. Epigyne (Fig. 8A, B) longer than wide, with broad fold anteriorly; epigynal window almost round, located posterior to the fold; copulatory ducts relatively short (less than the length of the receptacles), stout, expanding medially, connected to the inner anterior edge of the receptacles; receptacles oval, touching each other.

##### Distribution.

China (Yunnan, Guangxi).

##### Comments.

Although “*Gelotia* sp. [Guangxi] (from China)” of Maddison et al. (2014) is known by the figure of only the male palpal tibia, the structure is identical to *G. liuae* sp. nov. Thus, they are determined to be the same species, and the distribution of the new species includes Guangxi Province.

#### Gelotia zhengi

Taxon classificationAnimaliaAraneaeSalticidae

Cao & Li, 2016

40AF0770-A6D9-517E-814A-17A791DBD31C

**Figure 9.**
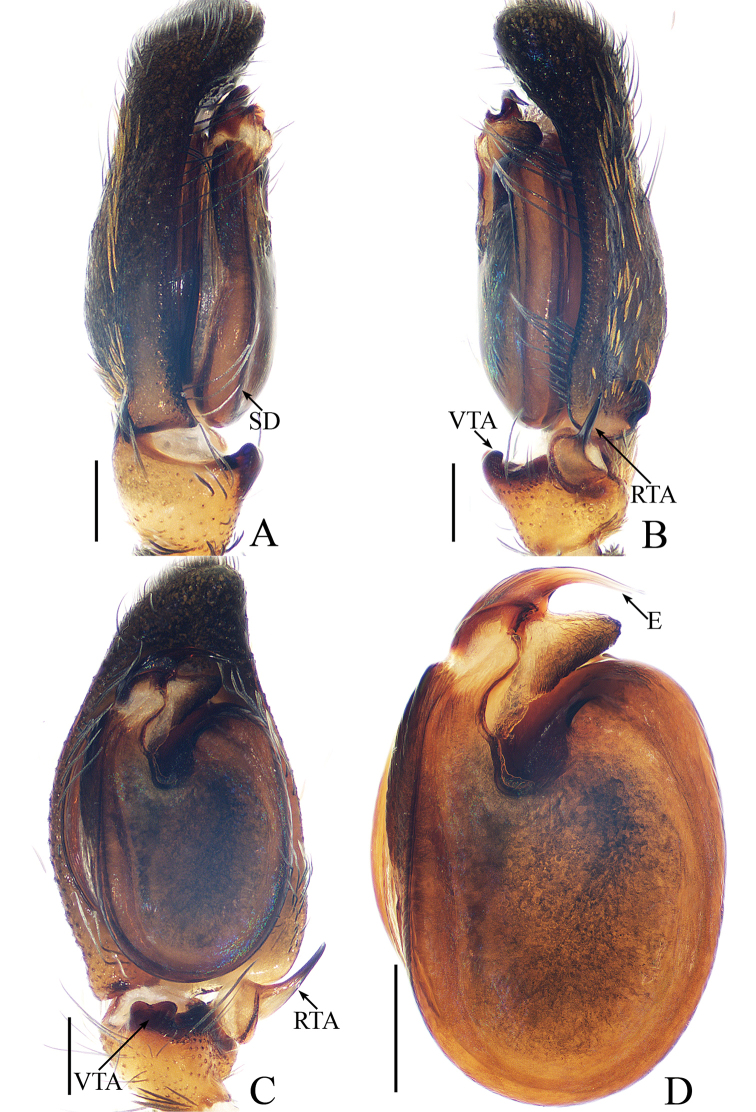
Male palp of *Gelotia zhengi* Cao & Li, 2016. **A** prolateral **B** retrolateral **C** ventral **D** bulb, retrolateral. Scale bars: 0.2.

**Figure 10.**
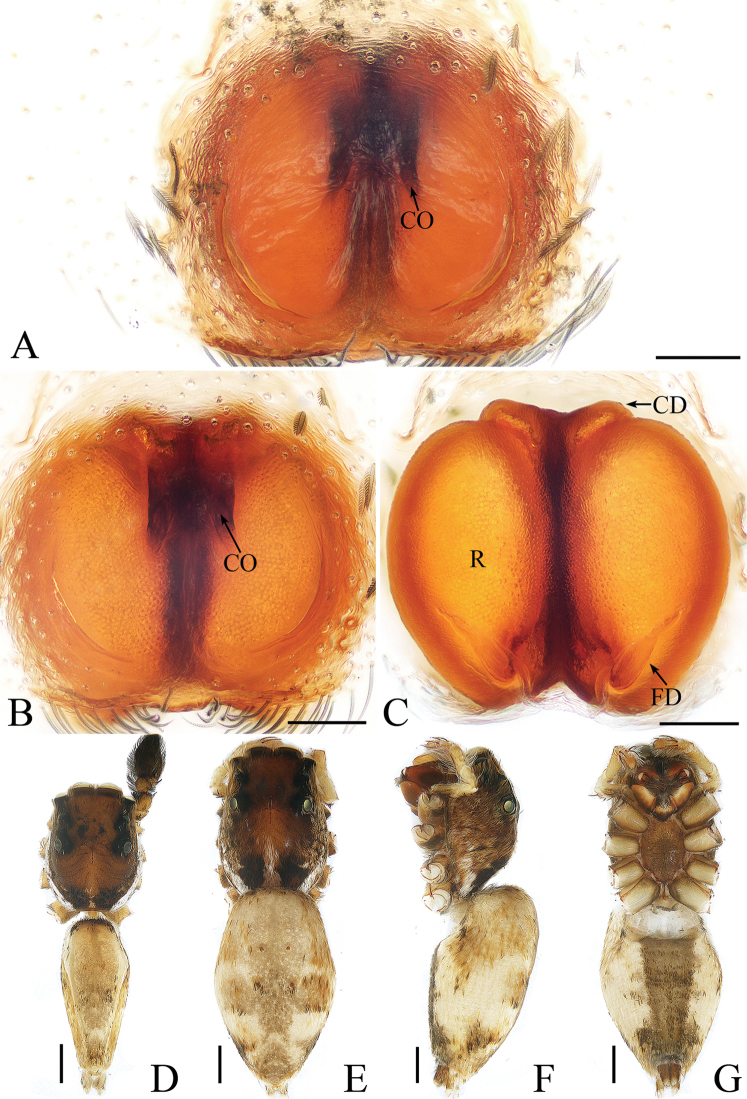
*Gelotia zhengi* Cao & Li, 2016. **A**, **B** epigyne, ventral **C** epigyne, dorsal **D** male habitus, dorsal **E** female habitus, dorsal **F** female habitus, lateral **G** female habitus, ventral. Scale bars: 0.1 (**A**–**C**); 1.0 (**D**–**G**).

Figs 9 , 10 , 17G , 18G , 19G

Gelotia zhengi Cao & Li, in Cao et al. 2016: 78, figs 24A–D, 25A, B (♂).

##### Material examined.

1♂ 1♀ (IZCAS Ar 39795–39796), CHINA: Yunnan: Xishuangbanna, Mengla County, Menglun Town, Menglun Nature Reserve, Lvshilin Rainforest Park, limestone tropical seasonal rainforest (21°54.58'N, 101°16.50'E, ca 570 m), 27.04.2019, C. Wang leg; 1♀ (IZCAS Ar 39797), Leprosy Village (21°53.62'N, 101°18.25'E, ca 520m), 4.05.2019, Y.F. Tong et al. leg.

##### Diagnosis.

The male has been diagnosed by Cao and Li (2016). The female resembles. *G. bimaculata* Thorell, 1890 from Borneo but differs by the following: 1) the receptacles are widest medially (Fig. 10C), whereas they are widest basally in *G. bimaculata* (Prószyński and Deeleman-Reinhold 2012, fig. 54); 2) the copulatory openings are situated medially (Fig. 10A, B), whereas they are situated anteriorly in *G. bimaculata* (Prószyński and Deeleman-Reinhold 2012, fig. 53).

##### Description.

***Male*.** Described by Cao and Li (2016).

***Female*.** Total length 9.13. Carapace 4.12 long, 2.97 wide. Abdomen 5.56 long, 3.38 wide. Clypeus 0.14 high. Eye sizes and inter-distances: AME 0.70, ALE 0.41, PLE 0.38, AERW 2.38, PERW 2.25, EFL 1.63. Legs: I 8.39 (2.39, 3.12, 1.90, 0.98), II 7.42 (2.12, 2.71, 1.71, 0.88), III 6.95 (1.95, 2.39, 1.78, 0.83), IV 8.29 (2.39, 3.12, 2.80, 0.98). Carapace (Figs 10E–G, 17G) red-brown, covered with dense brown setae, posteriorly with white stripes of setae. Clypeus yellow to brown, covered with several long setae. Chelicerae (Fig. 18G) red-brown, with 3 promarginal and 6 retromarginal teeth. Endites brown. Labium covered with dark setae. Sternum colored as endites, covered with brown setae. Legs yellow to brown, tibia of legs I with long, dark, dense setae ventrally. Spination of leg I: femur d1-1-3; tibia v2-2-2; metatarsus v2-0-2. Abdomen (Fig. 10E–G) elongated oval, dorsum with 2 pairs of muscle depressions medially, covered with dense yellow-brown setae, transverse white stripes postero-medially; venter with broad longitudinal brown stripe extending over the entire length, covered with brown setae. Epigyne (Fig. 10A–C) slightly wider than long; windows large, almost round; copulatory openings separated from each other by about 2 times their width, located medially; copulatory ducts stout, ascending before extending transversely to connect with long, oval receptacles; fertilization ducts lamellar.

##### Distribution.

China (Yunnan).

#### Irura

Taxon classificationAnimaliaAraneaeSalticidae

Peckham & Peckham, 1901

358C7372-B92F-5144-AB31-1D54E777500B

##### Type species.

*Irura pulchra* Peckham & Peckham, 1901 from Sri Lanka.

##### Comments.

The genus *Irura* is represented by 16 nominal species that are endemic to Vietnam (2), Malaysia (1), Sri Lanka (1), and China (11). The type locality of *I. mandarina* Simon, 1903 is unknown other than “Southeast Asia” (WSC 2020). The genus is rather poorly studied. Five species, including the generotype, are known from only females and two species from only males. The generotype, *I. pulchra*, is the westernmost species, and all other species are known from more than 2000 km east. The concept of the genus suggested by Peng et al. (1993) is followed here.

#### Irura lvshilinensis sp. nov.

Taxon classificationAnimaliaAraneaeSalticidae

A1F5435D-2C48-51C7-A33B-4ECA31E4B197

http://zoobank.org/23C20C31-C56C-439C-8DC2-E696834ACDE7

**Figure 11.**
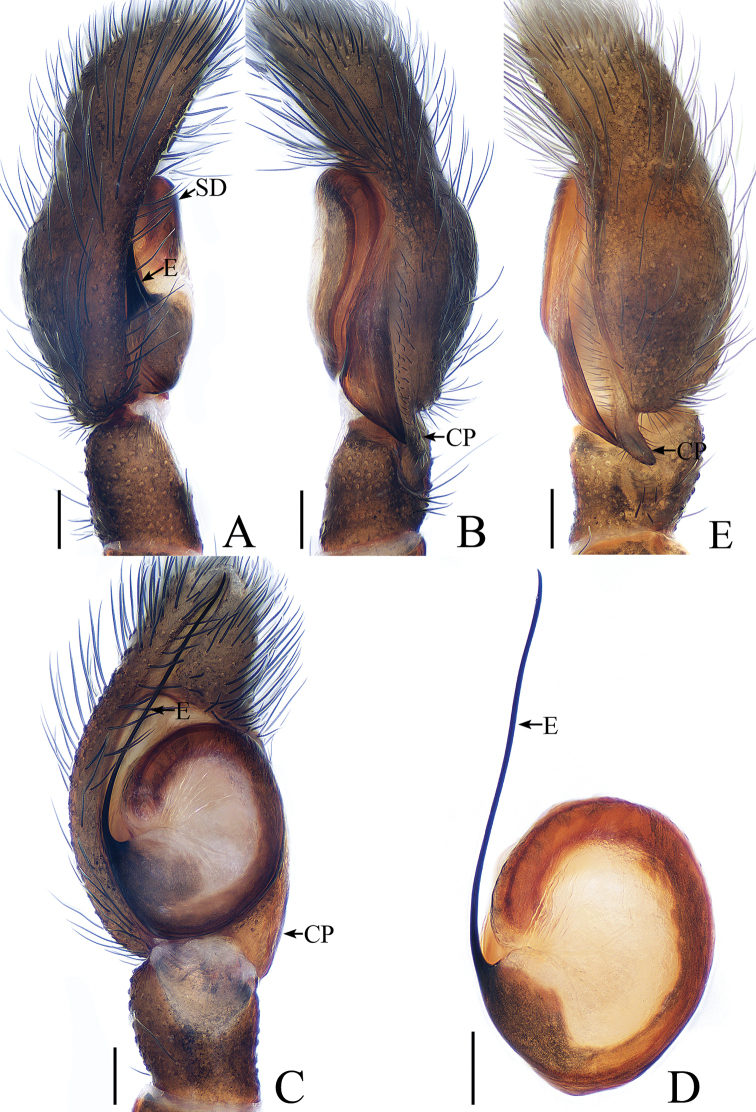
Male palp of *Irura lvshilinensis* sp. nov., **A**–**C**, **E** male holotype; **D** male paratype. **A** prolateral **B** retrolateral **C** ventral **D** bulb, ventral **E** dorsal. Scale bars: 0.1.

**Figure 12.**
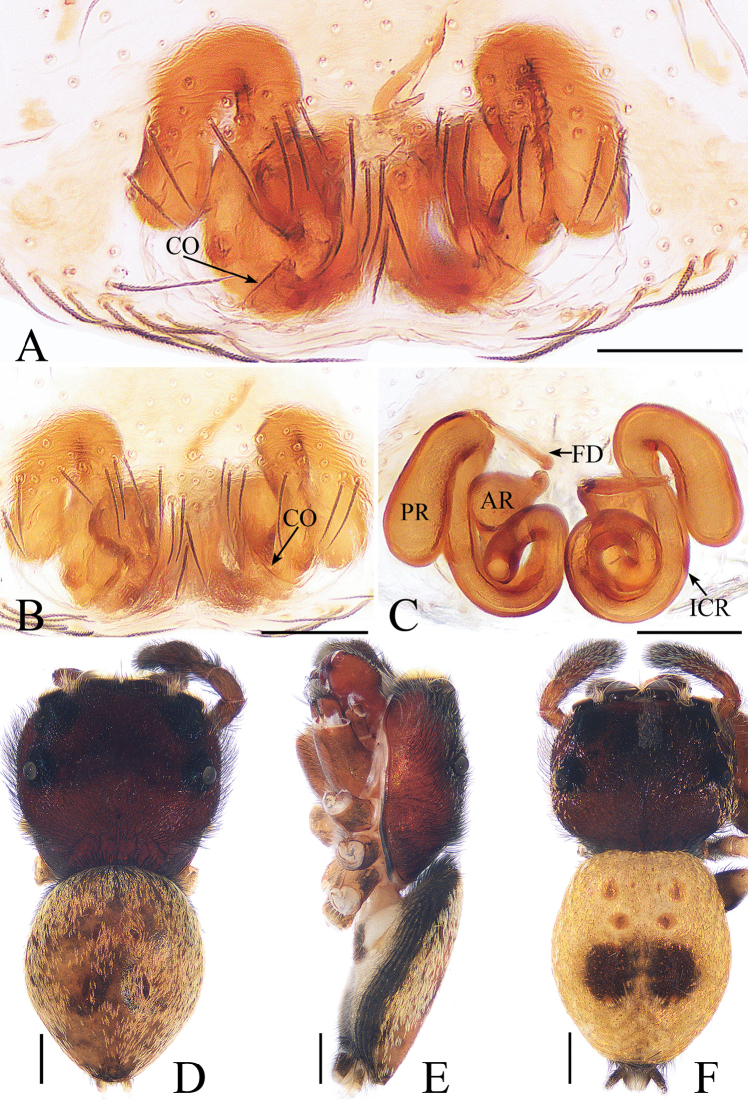
*Irura lvshilinensis* sp. nov., female paratype and male holotype. **A**, **B** epigyne, ventral **C** epigyne, dorsal **D** holotype habitus, dorsal **E** holotype habitus, lateral **F** female paratype habitus, dorsal. Scale bars: 0.1 (**A**–**C**); 0.5 (**D**–**F**).

Figs 11 , 12 , 17E , 18E , 19E

##### Type material.

***Holotype*** ♂ (IZCAS Ar 39798), CHINA: Yunnan: Xishuangbanna, Mengla County, Menglun Town, Menglun Nature Reserve, Lvshilin Rainforest Park, limestone tropical seasonal rainforest (21°54.58'N, 101°16.50'E, ca 570 m), 27.04.2019, C. Wang leg. ***Paratypes***: 2♂ 1♀ (IZCAS Ar 39799–39801), same data as holotype.

##### Etymology.

The species name is derived from the name of the type locality; adjective.

##### Diagnosis.

The male of *I. lvshilinensis* sp. nov. can be easily distinguished from other species considered in this genus except for *I. uniprocessa* Mi & Wang, 2016 from China by lacking a RTA. It can be distinguished from *I. uniprocessa* by the following: 1) the embolus is about one and a third times the bulb length (Fig. 11C, D), whereas it is almost equal to the bulb length in *I. uniprocessa* (Mi and Wang 2016, figs 1c, 2a); 2) the cymbial process extends distally above the tibia (Fig. 11B), whereas it does not extend beyond the tibia-cymbium joint in *I. uniprocessa* (Mi and Wang 2016, figs 1d, 2b). The female of the new species resembles *I. yunnanensis* (Peng & Yin, 1991,) known from China, in the general shape of the epigyne but differs in the following: 1) the intermediate canal of the receptacles is coiled and forms a loop medially (Fig. 12C), whereas the intermediate canal of the receptacles is only curved in *I. yunnanensis* (Peng and Yin 1991, fig. 3H); 2) the fertilization ducts are located medially (Fig. 12C), whereas they are located posteriorly in *I. yunnanensis* (Peng and Yin 1991, fig. 3H).

##### Description.

***Male*.** Total length 3.98. Carapace 1.96 long, 2.05 wide. Abdomen 2.20 long, 1.82 wide. Clypeus 0.03 high. Eye sizes and inter-distances: AME 0.45, ALE 0.29, PLE 0.21, AERW 1.68, PERW 1.95, EFL 0.96. Legs: I 5.00 (1.59, 2.24, 0.71, 0.46), II 2.99 (0.98, 1.07, 0.54, 0.40), III 2.73 (0.93, 0.90, 0.50, 0.40), IV 3.11 (1.02, 1.07, 0.61, 0.41). Carapace (Figs 12D, E, 17E) red-brown, covered with dense black setae, cephalic part with irregular dark stripe medially, thoracic part sloping acutely, with pair of dark stripes. Clypeus brown, with long setae. Fovea indistinct. Chelicerae (Fig. 18E) red-brown, with 2 promarginal teeth and 1 retromarginal fissident with 4 cusps. Endites yellow-brown, labium colored as endites, tip covered with dense, dark setae. Sternum yellow. Legs brown to red-brown; legs I stronger than others. Spination of leg I: tibia v0-2-2; metatarsus v2-0-2. Abdomen (Fig. 12D, E) oval, dorsum with 3 pairs of muscle depressions medially, covered with setae; venter pale, with large, brown markings posteriorly. Palp (Fig. 11A–E): femur red-brown, about 3.5 times longer than wide; patella colored as femur, slightly longer than wide; tibia slightly longer than wide, lacking apophysis; cymbium flattened, longer than wide, proximo-retrolaterally with well-developed process extending above tibia about 1/5 tibial length in retrolateral view; bulb almost round, with sperm duct extending along margin; embolus slender, about 1.3 times bulb length, arising at almost 9 o’clock, with a pointed tip that reaches cymbial tip.

***Female*.** Total length 3.65. Carapace 1.54 long, 1.67 wide. Abdomen 2.14 long, 1.79 wide. Clypeus 0.03 high. Eye sizes and inter-distances: AME 0.37, ALE 0.21, PLE 0.19, AERW 1.39, PERW 1.74, EFL 0.88. Legs: I 3.63 (0.98, 1.63, 0.61, 0.41), II 2.64 (0.85, 0.95, 0.44, 0.40), III 2.34 (0.76, 0.78, 0.40, 0.40), IV 2.91 (0.95, 1.10, 0.46, 0.40). Habitus (Fig. 12F) similar to that of male except paler, with pair of dark round patches on the dorsum of the abdomen. Epigyne (Fig. 12A–C) slightly wider than long; copulatory openings located posteriorly; copulatory ducts indistinct; receptacle divided into 2 chambers interconnected by an intermediate canal, anterior chamber almost pyriform, posterior chamber elongated oval; intermediate canal of receptacles long, coiling into complete circle medially; fertilization ducts slender, situated medially.

##### Distribution.

China (Yunnan).

#### Rhene

Taxon classificationAnimaliaAraneaeSalticidae

Thorell, 1869

30A3F45D-A680-5839-918A-5F89BEACD2DB

##### Type species.

*Rhanis flavigera* C.L. Koch, 1846 from Indonesia.

##### Comments.

The genus *Rhene* with 64 named species has never been revised. Both sexes are not yet known for more than two-thirds (42) of the species, and some are known from juvenile specimens. To date, 19 species have been recorded from Southeast Asia. Of these, 10 are known from only a single sex: seven from males and three from females, and two species are known from juvenile specimens. Presently, 10 species, including five endemics, are known from China (WSC 2020).

#### Rhene menglunensis sp. nov.

Taxon classificationAnimaliaAraneaeSalticidae

44476568-1928-5447-8E81-6BE25BDC8F8E

http://zoobank.org/8132458E-6CCE-498C-BA89-11BF797FB534

**Figure 13.**
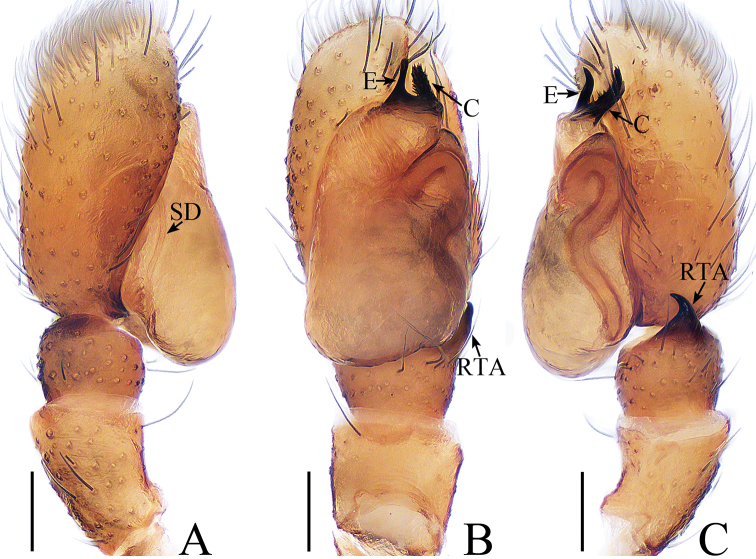
Male palp of *Rhene menglunensis* sp. nov., male holotype. **A** prolateral **B** ventral **C** retrolateral. Scale bars: 0.1.

**Figure 14.**
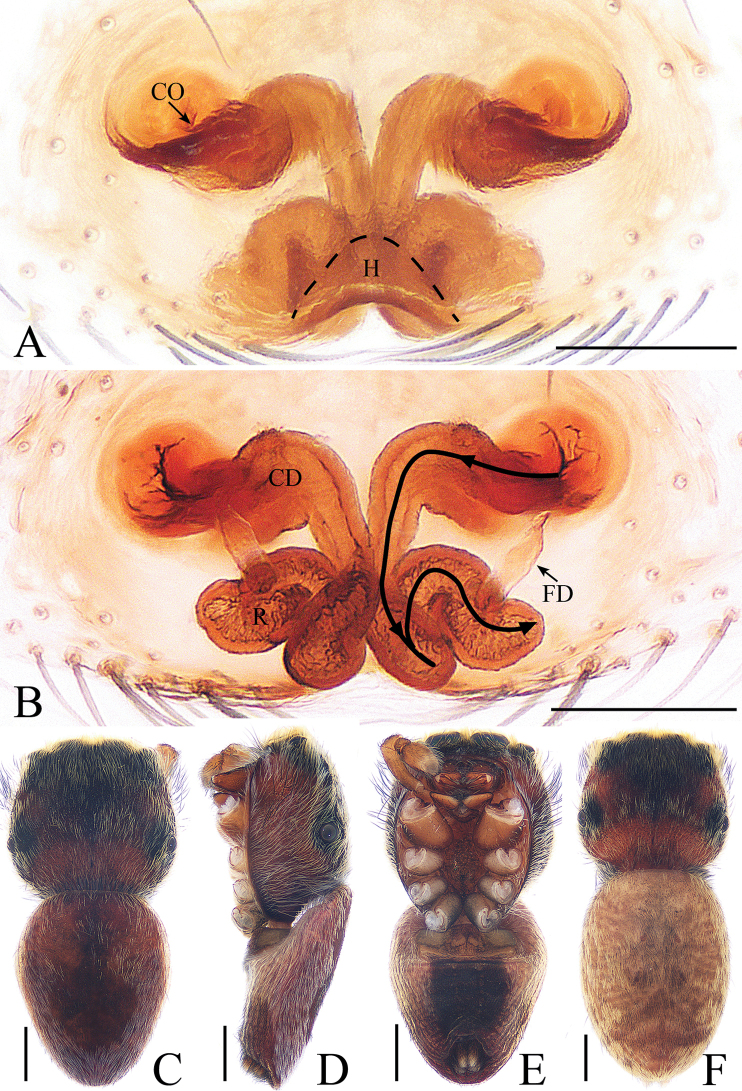
*Rhene menglunensis* sp. nov., female paratype and male holotype. **A** epigyne, ventral **B** epigyne, dorsal **C** holotype habitus, dorsal **D** holotype habitus, lateral **E** holotype habitus, ventral **F** female paratype habitus, dorsal. Scale bars: 0.1 (**A**, **B**); 0.5 (**C**–**F**).

Figs 13 , 14 , 17F , 18F , 19F

##### Type material.

***Holotype*** ♂ (IZCAS Ar 39802) CHINA: Yunnan: Xishuangbanna, Mengla County, Menglun Town, Menglun Nature Reserve, garbage dump, secondary tropical rainforest (21°54.30'N, 101°16.78'E, ca 620 m), 26.04.2019, Y.F. Tong et al. leg. ***Paratypes***: 3♀ (IZCAS Ar 39803–39805), same data as holotype; 2♂ 1♀ (IZCAS Ar 39806–39808), same locality, Vine Garden (21°55.80'N, 101°45.41'E, ca 550 m), 2.08.2018, C. Wang et al. leg; 1♂ (IZCAS Ar 39809), Lvshilin Rainforest Park, limestone tropical seasonal rainforest (21°54.58'N, 101°16.50'E, ca 570 m), 27.04.2019, C. Wang leg; 1♂ (IZCAS Ar 39810), same locality, tropical rainforest (21°55.20'N, 101°16.21'E, ca 550 m), 30.04.2019, Y.F. Tong et al. leg; 1♂ (IZCAS Ar 39811), riverside near the suspension bridge (21°56.02'N, 101°15.06'E, ca 550 m), 1.05.2019, C. Wang leg.

##### Etymology.

The species name is derived from the name of the type locality; adjective.

##### Diagnosis.

The male of *R. menglunensis* sp. nov. can be easily distinguished from other species of the genus by the conductor having several spinose processes. The female of the new species resembles *R. atrata* Karsch, 1881 known from Far East Asia in the general shape of the epigyne but differs by the following: 1) the epigynal hood is wider than long (Fig. 14A), whereas it is longer than wide in *R. atrata* (Logunov 1993, fig. 3C); 2) the fertilization ducts originate from the anterior part of the receptacles (Fig. 14B), whereas they originate from the posterior part of the receptacles in *R. atrata* (Logunov 1993, fig. 3D).

##### Description.

***Male*.** Total length 3.15. Carapace 1.47 long, 1.55 wide. Abdomen 1.82 long, 1.43 wide. Clypeus 0.04 high. Eye sizes and inter-distances: AME 0.36, ALE 0.20, PLE 0.15, AERW 1.22, PERW 1.58, EFL 0.99. Legs: I 3.14 (1.10, 1.20, 0.44, 0.40), II 2.38 (0.76, 0.80, 0.42, 0.40), III 2.19 (0.68, 0.71, 0.40, 0.40), IV 2.74 (0.88, 0.95, 0.51, 0.40). Carapace (Figs 14C–E, 17F) red-brown, with irregular dark stripe antero-medially, covered with dense grey-white setae. Clypeus red-brown, covered with dark, long setae. Chelicerae (Fig. 18F) red-brown, with 1 retromarginal tooth and 2 promarginal teeth. Endites, labium, and sternum colored as chelicerae. Legs I robust, red-brown, other legs pale yellow. Spination of leg I: femur d0-0-1, p0-0-3; tibia v0-0-2; metatarsus v2-0-2. Abdomen (Fig. 14C–E) elongated oval, with a pattern of darker setae medially, covered with dense setae; venter red to dark brown, without distinct markings. Palp (Fig. 13A–C): femur yellow, about 3 times longer than wide; patella colored as femur, almost as long as wide; tibia wider than long, with claw-shaped RTA shorter than tibia, tapering distally and curved towards bulb medially; cymbium longer than wide, slightly longer than the length of the bulb in retrolateral view; bulb longer than wide, with sperm duct extending along margin; embolus short, blunt apically in ventral view; conductor relatively thick, with spinose processes.

***Female*.** Total length 3.74. Carapace 1.53 long, 1.65 wide. Abdomen 2.32 long, 1.62 wide. Clypeus 0.04 high. Eye sizes and inter-distances: AME 0.35, ALE 0.19, PLE 0.15, AERW 1.19, PERW 1.58, EFL 0.96. Legs: I 2.84 (1.15, 1.05, 0.32, 0.32), II 2.34 (0.80, 0.88, 0.34, 0.32), III 2.18 (0.71, 0.73, 0.42, 0.32), IV 2.96 (0.98, 1.12, 0.54, 0.32). Habitus (Fig. 14F) similar to that of male except paler and lacking a clear pattern. Epigyne (Fig. 14A, B) with distinct posterior hood wider than long; copulatory openings almost cambered, situated anteriorly; copulatory ducts long, widest at base; fertilization ducts knife-shaped.

##### Distribution.

China (Yunnan).

#### Siler

Taxon classificationAnimaliaAraneaeSalticidae

Simon, 1889

85B9DAAE-4A67-59F1-ADD5-3F2DF5443EC5

##### Type species.

*Siler cupreus* Simon, 1889 from Japan.

##### Comments.

The genus *Siler* contains 10 nominal species currently known from East, South, and Southeast Asia. It is rather poorly studied and has not been revised. More than half (six) of the species are known from only a single sex: four from males and two from females. Additionally, one species has never been illustrated. Five species, including an endemic, occur in China (WSC 2020).

#### Siler zhangae sp. nov.

Taxon classificationAnimaliaAraneaeSalticidae

7029BFDF-D3B8-5979-BFB2-1AB7883519C5

http://zoobank.org/C6DDC2DE-468F-4CF2-BE43-D1AA19FB82CC

**Figure 15.**
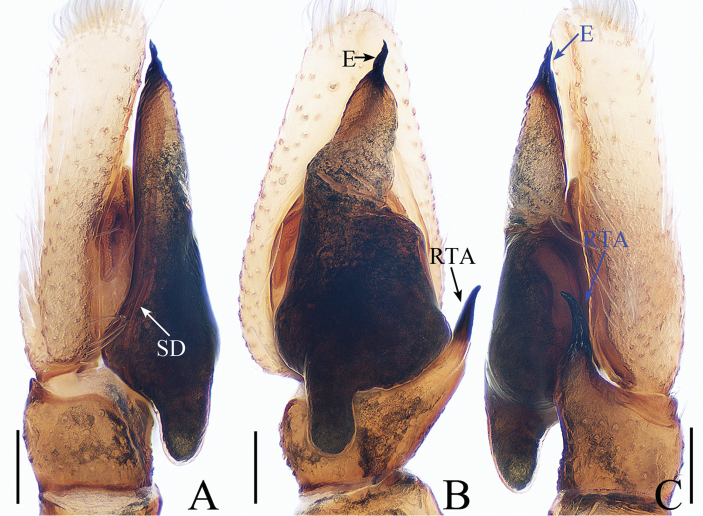
Male palp of *Siler zhangae* sp. nov., male holotype. **A** prolateral **B** ventral **C** retrolateral. Scale bars: 0.1.

**Figure 16.**
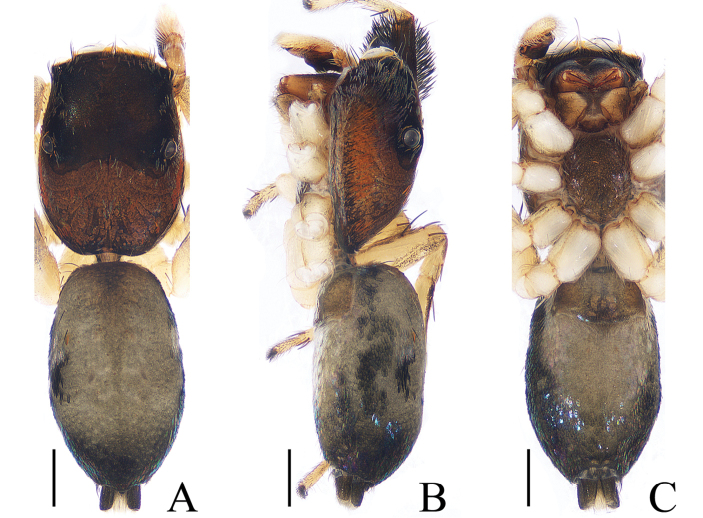
Habitus of *Siler zhangae* sp. nov., male holotype. **A** dorsal **B** lateral **C** ventral. Scale bars: 0.5.

**Figure 17.**
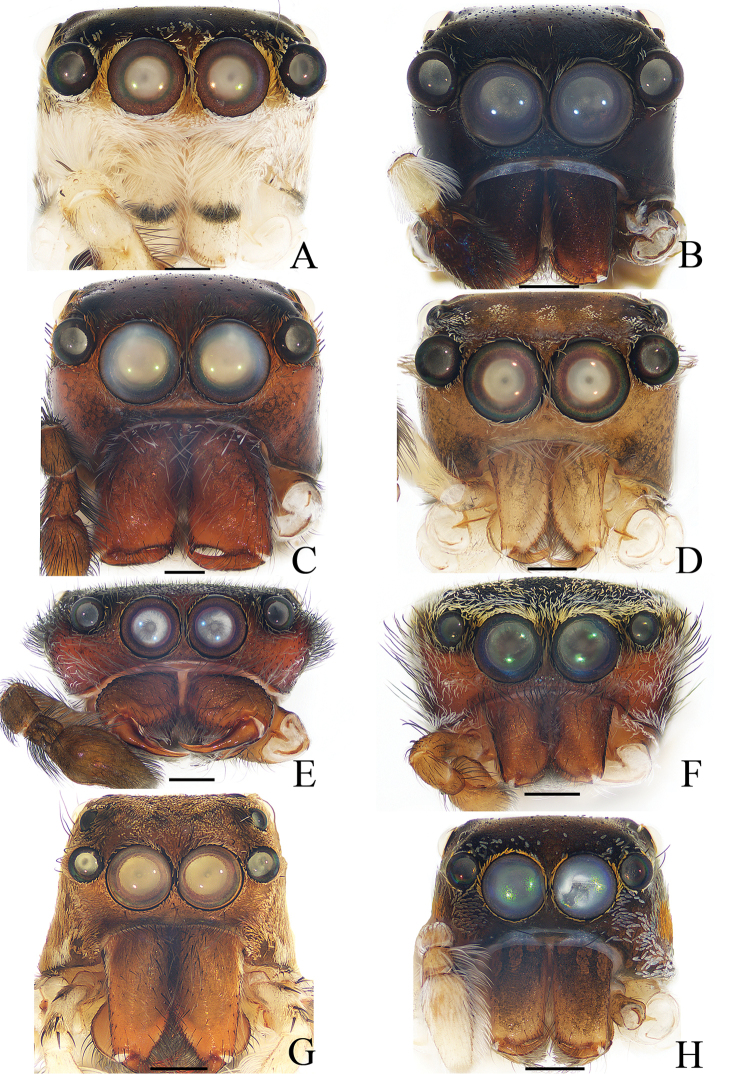
Frontal view of Carapace, **A–F, H** male holotype; **G** female. **A***Cytaea tongi* sp. nov. **B***Euophrys subwanyan* sp. nov. **C***Dexippus pengi* sp. nov. **D***Gelotia liuae* sp. nov. **E***Irura lvshilinensis* sp. nov. **F***Rhene menglunensis* sp. nov. **G***Gelotia zhengi* Cao & Li, 2016 **H***Siler zhangae* sp. nov. Scale bars: 0.3 (A–F, H); 0.5 G.

**Figure 18.**
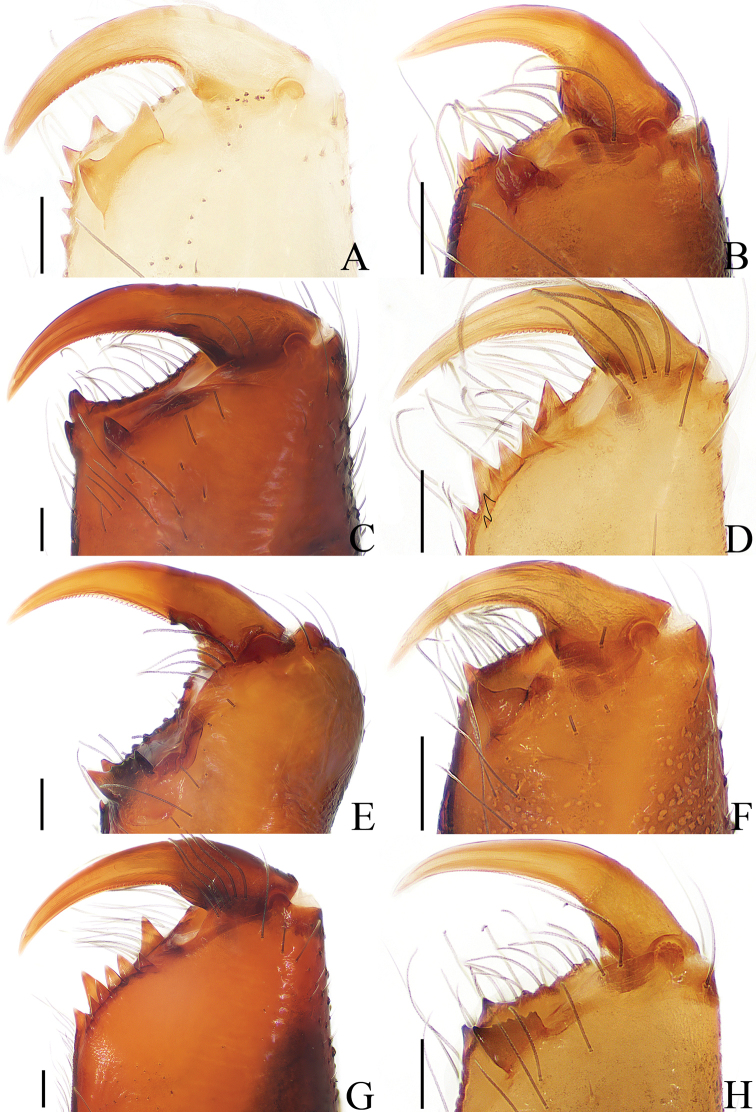
Dorsal view of chelicerae, **A**–**F**, **H** male holotype; **G** female. **A***Cytaea tongi* sp. nov. **B***Euophrys subwanyan* sp. nov. **C***Dexippus pengi* sp. nov. **D***Gelotia liuae* sp. nov. **E***Irura lvshilinensis* sp. nov. **F***Rhene menglunensis* sp. nov. **G***Gelotia zhengi* Cao & Li, 2016 **H***Siler zhangae* sp. nov. Scale bars: 0.1.

**Figure 19.**
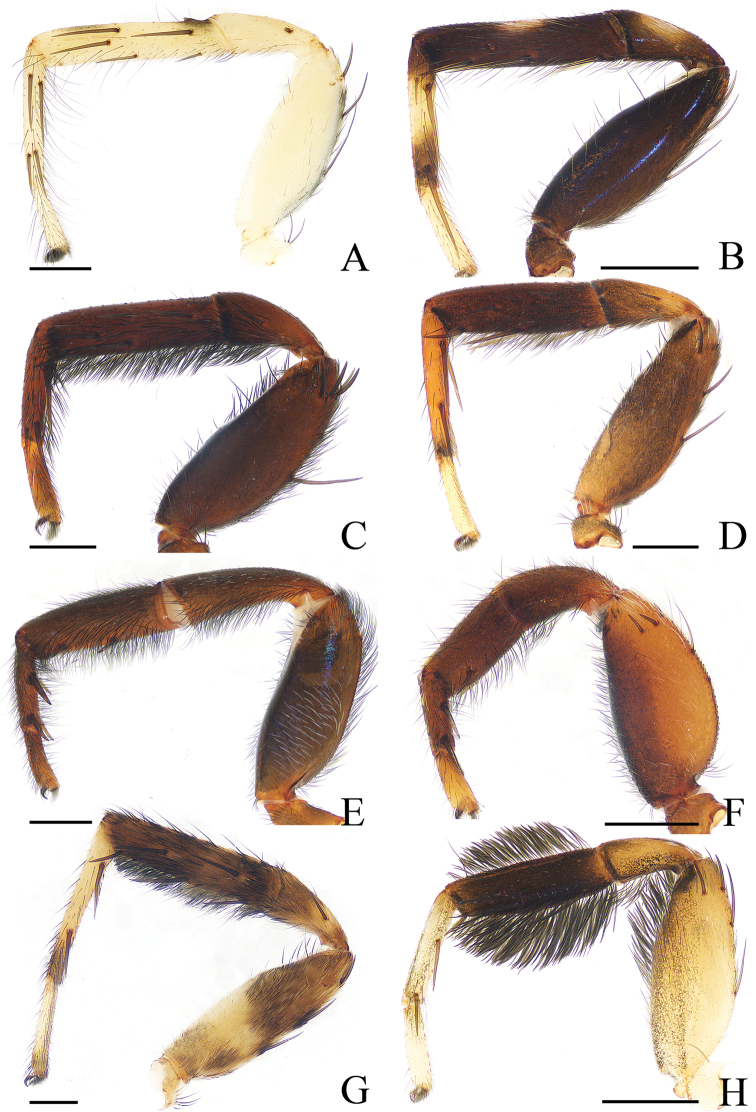
Prolateral view of right leg I, **A**–**F**, **H** male holotype; **G** female. **A***Cytaea tongi* sp. nov. **B***Euophrys subwanyan* sp. nov. **C***Dexippus pengi* sp. nov. **D***Gelotia liuae* sp. nov. **E***Irura lvshilinensis* sp. nov. **F***Rhene menglunensis* sp. nov. **G***Gelotia zhengi* Cao & Li, 2016 **H***Siler zhangae* sp. nov. Scale bars: 0.5.

Figs 15 , 16 , 17H , 18H , 19H

##### Type material.

***Holotype*** ♂ (IZCAS Ar 39819), CHINA: Yunnan: Xishuangbanna, Mengla County, Menglun Town, Menglun Nature Reserve, Lvshilin Rainforest Park, limestone tropical seasonal rainforest (21°54.58'N, 101°16.50'E, ca 570 m), 6.08.2018, C. Wang et al. leg. **Paratypes**: 2♂ (IZCAS Ar 39820–39821), same locality, Grapefruit Garden (21°54.07'N，101°16.36'E, ca 540 m), 25.07.2018, X.Q. Mi et al. leg; 1♂ (IZCAS Ar 39822), same locality, tropical rainforest (21°55.40'N, 101°16.32'E, ca 580 m), 11.08.2018, C. Wang et H. Liu leg.

##### Etymology.

The specific name is a patronym in honor of Dr Junxia Zhang (Baoding, China), who has contributed greatly to the taxonomy of jumping spiders worldwide.

##### Diagnosis.

*Siler zhangae* sp. nov. resembles *S. semiglaucus* (Simon, 1901) from Southeast Asia by having a relatively long bulb but differs by the following: 1) the embolus is directed anteriorly (Fig. 15B), whereas it is directed antero-prolaterally in *S. semiglaucus* (Peng et al. 1993, fig. 747); 2) the posterior lobe of the bulb is blunt (Fig. 15B), whereas it is pointed in *S. semiglaucus* (Peng et al. 1993, fig. 747); 3) the embolus is twisted (Fig. 15B, C), whereas it is not twisted in *S. semiglaucus* (Peng et al. 1993, figs 747, 748).

##### Description.

***Male*.** Total length 3.76. Carapace 1.68 long, 1.27 wide. Abdomen 1.98 long, 1.19 wide. Clypeus 0.04 high. Eye sizes and inter-distances: AME 0.36, ALE 0.21, PLE 0.16, AERW 1.08, PERW 1.19, EFL 0.86. Legs: I 3.82 (1.32, 1.41, 0.68, 0.41), II 2.91 (0.93, 1.02, 0.56, 0.40), III 3.29 (1.02, 1.10, 0.73, 0.44), IV 4.31 (1.29, 1.51, 1.07, 0.44). Carapace (Figs 16A–C, 17H) red-brown, widest between coxae II and III, covered with white scale-like setae. Clypeus dark brown. Fovea longitudinal. Chelicerae (Fig. 18H) yellow, with 2 promarginal teeth and 1 retromarginal fissident. Endites widest at tip. Sternum brown, covered with thin setae. Tibia of legs I with characteristic brushes of long, dark, dense setae ventrally and dorsally. Spination of leg I: femur d1-1-3; tibia v2-0-2; metatarsus v0-2-2. Abdomen (Fig. 16A–C) elongated oval, dorsum with 2 pairs of muscle depressions, and scale-shaped setae bilaterally and posteriorly; venter pale brown, also with scale-shaped setae. Palp (Fig. 15A–C): femur yellow, about 3 times longer than wide, covered with dense white setae; patella colored as femur, almost as long as wide; tibia wider than long, with RTA tapering toward the tip, slightly longer than tibia, tip slightly bent ventrally; cymbium flattened, widest at base, tapering in ventral view; bulb elongated, widest at base, with well-developed, blunt posterior lobe extending above tibia about half the length of the tibia in ventral view; embolus longer than RTA with long, subconical base, and relatively short, twisted tip directed anteriorly.

***Female*.** Unknown.

##### Distribution.

China (Yunnan).

##### Comments.

This species is described based on males only, and so there is a possibility it is conspecific to one of the two species (*S. flavocinctus* (Simon, 1901), *S. bielawskii* Zabka, 1985) known from only females.

## Supplementary Material

XML Treatment for Cytaea

XML Treatment for Cytaea tongi

XML Treatment for Dexippus

XML Treatment for Dexippus pengi

XML Treatment for Euophrys

XML Treatment for Euophrys subwanyan

XML Treatment for Gelotia

XML Treatment for Gelotia liuae

XML Treatment for Gelotia zhengi

XML Treatment for Irura

XML Treatment for Irura lvshilinensis

XML Treatment for Rhene

XML Treatment for Rhene menglunensis

XML Treatment for Siler

XML Treatment for Siler zhangae
